# 25-Hydroxyvitamin D assay standardisation and vitamin D guidelines paralysis

**DOI:** 10.1017/S1368980019005251

**Published:** 2020-05

**Authors:** CT Sempos, N Binkley

**Affiliations:** 1 Vitamin D Standardization Program LLC, Havre de Grace, MD, USA Email semposch@gmail.com; 2 Osteoporosis Clinical Research Program and Institute on Aging, Department of Medicine, University of Wisconsin-Madison, Madison, WI, USA

## Overview: vitamin D guidelines paralysis

**Fig. 1:**
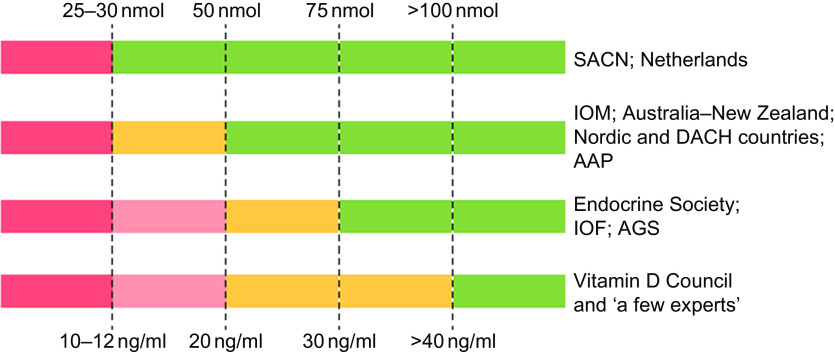
Recommendations for interpreting serum levels of 25-hydroxyvitamin D. ‘A schematic representation of how different agencies and countries interpret serum levels of 25-hydroxyvitamin D is shown. Colour code: red denotes a state of severe deficiency (danger) that has to be corrected without exception; orange denotes a state of mild deficiency (modest concern), in which intervention is desirable; green denotes a state of sufficient supply that does not benefit from additional supplementation. AAP, American Academy of Pediatrics; AGS, American Geriatrics Society; DACH, Deutschland (Germany, Austria and Confoederatio Helvetica (Switzerland); IOF, International Osteoporosis Foundation; IOM, Institute of Medicine; SACN, Scientific Advisory Committee on Nutrition.’ Source: Bouillon^(1)^

Vitamin D guidelines development is in a state of paralysis. There is an outward sign of a growing consensus between the two groups of nations: (1) the UK and the Netherlands with (2) Australia–New Zealand, European Union (EU) and the USA in how to define the lower limit of vitamin D adequacy based on serum total 25-hydroxyvitamin D (25(OH)D) concentration^(1)^ (Fig. 1), that is, the sum of serum 25(OH)_2_ and 25(OH)D_3_ concentrations. But the two groups do not agree if there are data to support defining additional vitamin D physiological states, e.g. *insufficiency*, *sufficiency* and *toxicity* based on 25(OH)D concentrations. Moreover, those national recommendations conflict with a third set of recommendations from non-governmental medical societies and organisations^(1)^, for example, the Endocrine Society, with no prospect for resolving the differences among the three approaches^(1,2)^. Given the wealth of vitamin D research, it could be expected that the controversy would be resolved and consensus reached. Why has this not occurred?

To understand this situation, we need to understand the requirements for making and revising guidelines and policies that result from those guidelines. Understanding those requirements leads to a rather straightforward conclusion: Making and revising guidelines requires 25(OH)D data from rigorously conducted research and nationally representative surveys^(3,4)^. In addition, nationally representative survey data are required to accurately and precisely assess the current state of vitamin D status in the population by person, season and geographical location, and to monitor changes over time in order to develop rational guidelines and associated policies and revise them over time^(5,6)^. But variability of 25(OH)D assays thwarts attempts to resolve the controversy^(7)^.

Variability of 25(OH)D assays is widely recognised^(7–10)^. As such, only standardised 25(OH)D data provide the necessary level of accuracy and precision essential to the process of developing evidence-based vitamin D guidelines and policies^(3–6)^. In the last 10 years, enormous progress has been made in collecting nationally representative survey data that meet those requirements^(11–16)^, but despite this progress, we remain woefully behind in generating the vitamin D research data necessary to break out of the paralysis.

Thus, we have at a minimum three basic approaches for defining different states of vitamin D status, and no way to determine which is most appropriate. The goals of this Commentary are to describe the origins of the problem and to propose a set of recommendations based on work of the Vitamin D Standardization Program (VDSP) that may provide a way forward in developing rational vitamin D status guidelines.

## Origin of vitamin D guidelines, 25(OH)D assay variability and policy paralysis

**Fig. 2:**
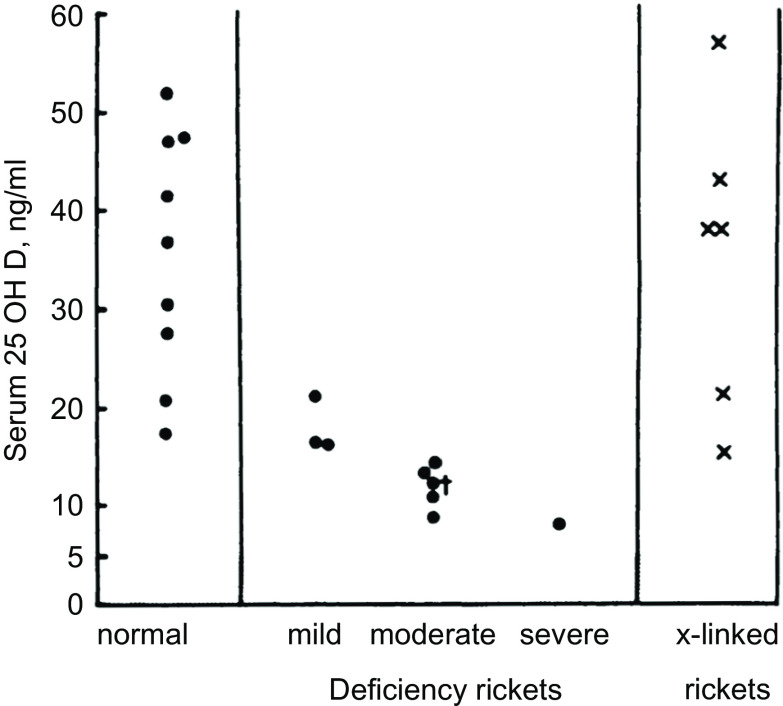
Distribution of serum 25-hydroxyvitamin D values in normal and rachitic children, aged 2 months–6 years (Source: Arnaud *et al*.^(18)^) *‘Patient No. 7 represents 25-OH-D; this patient had received 400 IU of vitamin D2 daily’.

The UK appears to be the first country to have adopted serum 25(OH)D cut-points for defining vitamin D status. In 1991, the UK Committee on Medical Aspects of Food and Nutrition Policy (COMA) Dietary Reference Values (DRV) report stated that ‘Plasma 25-OHD concentrations in rickets range from not detectable to about 8 ng/ml’^(17)^. That appears to be the first recommended cut-off for 25(OH)D. The quoted source was a 1976 paper by Arnaud *et al*.^(18)^ reporting serum 25(OH)D concentrations ranging between approximately 8 and 20 ng/ml in nine cases of rickets (Fig. 2). As a result, it appears that the UK DRV 1991 Committee acted conservatively in picking an 8 ng/ml (20 nmol/l) 25(OH)D cut-point. In the 1998 UK COMA report, the 25(OH)D cut-point was raised to 10 ng/ml (25 nmol/l)^(19)^. In this case, the quoted source was a 1986 paper by Grindulis *et al*.^(20)^. It is not clear why the value was increased to 10 ng/ml (25 nmol/l), but it may have been as simple as 10 is a round number easy for physicians to remember. The recent 2016 UK Scientific Advisory Committee on Nutrition (SACN) retained the 1998 COMA 10 ng/ml (25 nmol/l) concentration to define the lower limit of adequacy, ‘based on evidence suggesting risk of rickets and osteomalacia is increased at concentrations below this level’^(21)^. Notably, variability within and among 25(OH)D assays was cited as an important limitation ‘for interpretation of studies that have examined the relationship between serum 25(OH)D concentration and health outcomes’.^(21)^.

**Fig. 3:**
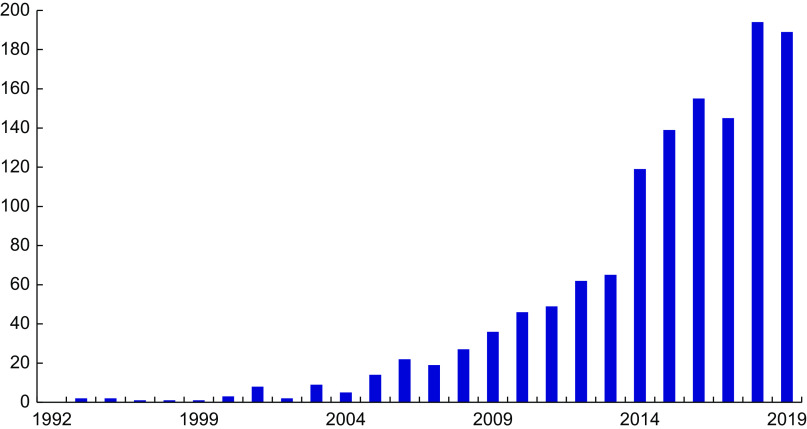
Vitamin D meta-analyses published since 1992. Source: PubMed: https://www.ncbi.nlm.nih.gov/pubmed/ (accessed September 2019)

Concern about 25(OH)D assay variability is not a new problem. The problem was first reported in 1983–1984^(22–24)^ and in many publications since that time^(25,26)^. As a result, the Vitamin D External Quality Assessment Scheme (DEQAS) was introduced in 1989 to improve the reliability of 25(OH)D assays^(26)^. In 2013, DEQAS became an accuracy-based external quality assessment scheme (EQAS) with Joint Committee for Traceability in Laboratory Medicine (JCTLM)-approved reference measurement procedures from the National Institute for Standards and Technology (NIST) and the Centers for Disease Control and Prevention (CDC) being used to provide the target values, that is, true 25(OH)D concentration, for serum samples used in DEQAS^(27,28)^.

Currently, there are primarily two basic types of assays in use: (1) automated immunoassays, which are commercially developed and marketed; and (2) chromatography-based assays, which are primarily laboratory-developed using HPLC or LC-MS/MS^(25,29,30)^. Trends in the performance of assays used by participating laboratories have been tracked since the initiation of DEQAS^(26)^.

Recent DEQAS results (January 2019) document that 25(OH)D assay variability persists among the ten assays most commonly used by participating laboratories (Table 1). Clearly, mean bias, defined as the percentage difference from the true concentration, varies by sample within an assay, and there is enormous variability around the mean for all the assays. Moreover, there is a great deal of variability among the different assays. HPLC and especially LC-MS/MS assays are often assumed to be the gold standard; however, results in Table 1 demonstrate that a great deal of variability exists among the laboratories using them. Similar results were found in the recent analysis of data from the College of American Pathologists (CAP) Accuracy-Based Vitamin D (ABVD) survey^(31)^. Thus, using a chromatography-based assay and participating in an external quality assessment programme does not assure that ‘25(OH)D’ results from research studies or national surveys are accurate^(31,32)^. This is an essential point: without documented assay standardisation, it *cannot* be assumed that just because a laboratory uses an HPLC or LC-MS/MS assay the results are either accurate or precise.

**Table 1:** Mean bias from the ‘true’ sample concentration for the ten most commonly used assay platforms of the laboratories participating in the Vitamin D External Quality Assessment Scheme (DEQAS)*

Assay	Labs (*n*)	DEQAS sample 550		DEQAS sample 546		DEQAS sample 549		DEQAS sample 547		DEQAS sample 548	
		CDC† 24·5 nmol/l		CDC† 52·1 nmol/l		CDC† 61·9 nmol/l		CDC† 71·3 nmol/l		CDC† 87·0 nmol/l	
		Mean bias (%)	95 % CI	Mean bias (%)	95 % CI	Mean bias (%)	95 % CI	Mean bias (%)	95 % CI	Mean bias (%)	95 % CI
Abbott Architect New	64	–7	–24, 9	–4	–15, 6	–2	–12, 7	–1	–12, 10	7	–3, 17
Bechman Unicel	35	6	–38, 50	–8	–31, 15	–11	–35, 12	–1	–22, 20	–2	–20, 17
DiaSorin Liaison Total	185	18	–4, 41	7	–10, 25	10	–6, 26	4	–12, 20	7	–9, 24
HPLC	17	7	–52, 66	–1	–41, 40	8	–64, 80	7	–42, 57	4	–37, 46
IDS-iSYS	31	0	–44, 44	5	–23, 33	2	–14, 18	–5	–25, 15	1	–16, 18
IDS-SYS New	17	4	–25, 33	6	–25, 37	7	–17, 30	5	–19, 28	7	–16, 30
LC-MS/MS	143	–1	–23, 21	0	–20, 20	1	–18, 20	1	–18, 20	2	–17, 21
Roche Total 25OHD	76	5	–24, 35	4	–16, 24	1	–17, 18	12	–11, 35	10	–10, 31
Roche Vitamin D Total II	62	6	–21, 33	–5	–19, 9	–2	–17, 13	–1	–13, 11	–2	–14, 10
Siemens ADVIA Centuar	48	34	–15, 83	12	–16, 40	8	–18, 33	4	–23, 31	6	–18, 31

* January 2019 distribution for samples 546–550. Results are displayed by lowest to highest serum 25-hydroxyvitamin D concentrations of DEQAS samples. Data source: DEQAS Laboratory Report for January 2019 distribution (http://www.deqas.org/).

† CDC reference measurement procedure *true* concentration target value; Mineva *et al*.^(28)^.

Following the UK, the USA, in 1997, adopted a 25(OH)D concentration <11 ng/ml (27·5 nmol/ml) as the level consistent with vitamin D deficiency in infants, neonates and young children^(33)^. The quoted source was a paper by Specker *et al*.^(34)^. The US and Canada governments then co-sponsored an updated DRI review of calcium and vitamin D in 2011^(35)^. The 25(OH)D cut-off to define persons at risk of vitamin D deficiency was changed from <11 to <12 ng/ml (30 mnol/l), most likely to make it a round number to fit in with the IOM 2011’s definition of the 25(OH)D concentration consistent with both the average vitamin D requirement, that is, 16 ng/ml (40 nmol/l), and the requirements of approximately 98 % of the population, that is, 20 ng/ml (50 nmol/l). 25(OH)D cut-points were based on relationships to markers of bone health, and there was no single quoted source for the suggested cut-points.

Thus, the IOM 2011 guidelines were consistent with the UK guidelines and IOM 1997 in defining the lower limit of adequacy, but it was more expansive in setting 25(OH)D levels to define *inadequacy* 12–20 ng/ml (30–50 nmol/l), *sufficiency* 20–30 ng/ml (50–75 nmol/l), *no added benefit* 30–50 ng/ml (75–125 nmol/l) and *possible harm* >50 ng/ml (>125 nmol/l) such that we can speak of overlapping but non-congruent sets of national guidelines^(1)^ (Fig. 1). Moreover, the authors of the report commented that ‘Currently, different assays for the determination of serum 25OHD levels are in use, and they provide disparate results. In turn, reported measures are confounded by the need to understand the assay used and research reports contain results that are not easily compared. The role of standard reference materials and interlaboratory collaboration is an important aspect of overcoming the challenges that the assay methodologies present’^(35)^. In short, this report acknowledged that without assay standardisation, results from different studies are not comparable and, ideally, they should not be pooled to develop consensus results.

National committees like those in the UK and USA appropriately tend to be very conservative. Once guidelines have been established, they are re-evaluated only when there is sufficient new data^(36)^ and updated only when that data are irrefutable and there is consensus supporting change. No government agency wants to face a situation of having to retract one set of guidelines and replace it with a new set. With such a change, there is a loss of confidence in the entire process by the public at large, clinicians and scientists, all of which can result in the loss of necessary political support, that is, money, to develop and revise guidelines. Moreover, government laws, regulations, rules and programmes may be based on the guidelines, and changing and then unravelling existing guidance would not only result in lost confidence but an enormous cost in money and resources. As a result, once deficiency was defined in IOM 1997, it became the fulcrum around which future guidelines would revolve as seen in IOM 2011. An important issue that was hinted at in both IOM 2011 and SACN 2016 reports is that without assay standardisation further revision of 25(OH)D cut-points would be difficult, that is, paralysis.

Following the release of IOM 2011 guidelines, several other countries and medical societies released vitamin D guidelines (Fig. 1, Table 2). National agencies, more often than not, look to the guidelines of other countries when setting their own. The tendency is for national agencies to adopt the guidelines of other countries. In this case, the Nordic countries^(37)^, the Swiss Federal Commission on Nutrition^(38)^ along with the EFSA^(39)^ adopted IOM 2011 guidelines or IOM 2011-like guidelines for interpreting 25(OH)D concentrations, while the Netherlands, being a bit more conservative, adopted the UK 1998 COMA recommendations to define a 25(OH)D concentration of 10 ng/ml (25 nmol/l) as the level at which risk of rickets and osteomalacia increases for persons aged 0–70 years^(40)^. All these groups have adopted guidelines consistent with the conservative spirit of the UK DRV 1991 and IOM 2011 guidelines. That is, they define a serum 25(OH)D concentration of 25–30 nmol/l (10–12 ng/ml) as the lower limit of adequacy – an indicator of high risk of vitamin D deficiency (Fig. 1, Table 2). In addition, several organisations^(41–51)^, including the Global Consensus Recommendations on Prevention and Management of Nutritional Rickets^(51)^ (Table 2), have adopted guidelines consistent with IOM 2011 that define cut-points for insufficiency, sufficiency and possible harm. Does this indicate a developing consensus among health agencies? Possibly, but we believe it likely indicates conformity rather than evidence-based consensus.

**Table 2:** Selected* recommendations for interpreting serum total 25-hydroxyvitamin D concentrations by type of committee, year of publication and consistency with UK DRV 1991, IOM2011 or Endocrine Society 2012 recommendations

Type of committee	Year of publication	Consistent with
Governmental		
UK DRV^(17)^	1991	UK DRV 1991
US IOM^(33)^	1997	UK DRV 1991
UK COMA^(19)^	1998	UK DRV 1991
The Netherlands^(40)^	2012	UK DRV 1991†
UK SACN^(21)^	2016	UK DRV 1991
US IOM^(35)^	2011	US IOM 2011
NORDIC^(37)^	2012	US IOM 2011
Swiss Federal Commission for Nutrition^(38)^	2012	US IOM 2011
European Food Safety Authority^(39)^	2016	US IOM 2011
Non-governmental/medical societies		
Lawson Wilkins Pediatric Endocrine Society^(41)^	2008	US IOM 2011
German, Austrian, & Swiss Nutrition Societies (DACH)^(42)^	2012	US IOM 2011
Australia/New Zealand^(43,44)^	2012/2013	US IOM 2011
British Paediatric^(45)^	2012	US IOM 2011
French Society of Pediatrics^(46)^	2012	US IOM 2011
Spanish Association of Pediatrics^(47)^	2012	US IOM 2011
European Society for Paediatric Gastroenterology, Hepatology and Nutrition^(48)^	2013	US IOM 2011
American Academy of Pediatrics^(49,50)^	2008, 2014	US IOM 2011
Global Consensus Recommendations^(51)^	2016	US IOM 2011
Canadian Paediatric Society^(53)^	2007	Endocrine Society
International Osteoporosis Foundation^(54)^	2010	Endocrine Society
Scientific Advisory Council of Osteoporosis Canada^(55)^		
Endocrine Society^(52)^	2011	Endocrine Society
Central Europe^(56)^	2013	Endocrine Society
Society for Adolescent Health and Medicine^(57)^	2013	Endocrine Society
American Geriatrics Society Workshop^(58)^	2014	Endocrine Society
National Osteoporosis Foundation^(59)^	2014	Endocrine Society
Vitamin D Council^(60)^	2015	Endocrine Society
United Arab Emirates^(61)^	2016	Endocrine Society
Japanese Medical Societies^(62)^ †	2017	Endocrine Society‡
Italian Pediatric Society^(63)^	2018	Endocrine Society

* Adapted, in part, from Bouillon^(1)^ and Saggese *et al*.^(63)^.

† Expert panel supported by the research programme of intractable diseases; Ministry of Health, Labour and Welfare, Japan; the Japanese Society for Bone and Mineral Research; and the Japan Endocrine Society. It is not clear if this was a ‘governmental’ set of guidelines.

‡ Recommendations for: ages 0–70: serum 25(OH)D level >12 ng/ml (30 nmol/l); and age >70: risk of bone fractures and serum 25(OH)D > 20 ng/ml (50 nmol/l).

In 2011 a third distinct set of guidelines – ‘Evaluation, Treatment, and Prevention of Vitamin D Deficiency: An Endocrine Society *Clinical Practice* Guideline (emphasis added)’ – was released by the Endocrine Society^(52)^, and while they were quite similar to the 2007 guidelines released by the Canadian Paediatric Society^(53)^ (Table 2), they have come to be the focus of opposition to the UK DRV 1991 and IOM 2011 guidelines. Several medical societies and non-governmental organisations have adopted the Endocrine Society guidelines^(54–63)^. The Endocrine Society set the 25(OH)D concentration to define *deficiency*, *insufficiency*, *sufficiency* and *possible harm* at <20 ng/ml (50 nmol/l), 21–29 ng/ml (52·5–72·5), 30–100 ng/ml (75–250 nmol/l) and >100 ng/ml (>250 nmol/l), respectively^(52)^ based on the 2007 paper by Holick^(64)^.

The Endocrine Society guidelines were quite different from anything proposed by the UK DRV 1991 or IOM 2011 guidelines (Table 3). They set off a firestorm of debate between the authors of the Endocrine Society and the authors of IOM 2011 guidelines that has continued ever since.

**Table 3:** Comparison of Institute of Medicine^(35)^ *v*. Endocrine Society^(52)^ cut-points for serum total 25-hydroxyvitamin D (nmol/l)

Interpretation	IOM	Endocrine Society
	Serum total 25(OH)D (ng/ml)*	
Deficient	<12	<20
Insufficient	12–20	21–29
Sufficient	20–30	30–100
No added benefit	30–50	
Possible harm	>50	>100

* nmol/l = ng/ml × 2·5.

But there is even more confusion. The Endocrine Society stated that their guidelines were designed for clinical practice, while the IOM 2011 and later UK SACN 2016 demurred by stating that their guidelines were not for use in clinical practice but were public health guidelines for the general healthy non-diseased population^(21,35,52)^. We find it difficult to understand how a government agency can define interpretive guidelines for 25(OH)D – especially to define vitamin D deficiency – and not have them be clinically relevant. This emphasises the point that public health and clinical guidelines both need to revolve around identical sets of interpretive cut-points; otherwise there will be chaos and confusion among government agencies, physicians and the general public – as currently exists.

Given the sharp distinctions between the IOM 2011 and Endocrine Society guidelines, how can we go about determining the ‘best’ or ‘most appropriate’ cut-points given our current state of knowledge (Table 3)? Will meta-analyses solve the problem? There are an ever-increasing number published every year (Fig. 3), and yet we do not appear to be any closer to a resolution to this dilemma.

**Fig. 4:**
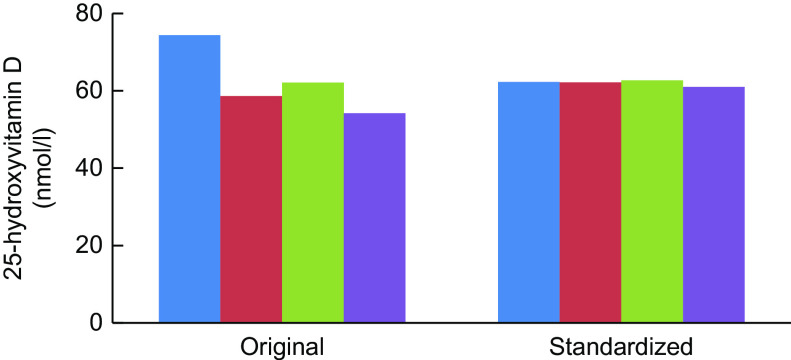
Trends in original assay and standardised mean 25-hydroxyvitamin D concentrations in nmol/l, USA, 1988–2006 (National Health and Nutrition Examination Surveys: 1988–1994, 2001–2002, 2003–2004 and 2005–2006. Survey-specific weighting factors were used to calculate representative means for the entire USA in each survey period. Standardised means were based on model 1 results. Please see source for more details. Source: Schleicher *et al*.^(14)^ (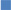, 1988–1994; 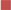, 2001–2002; 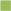, 2003–2004; 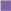, 2005–2006)

The fundamental reason why meta-analyses of the currently available data will not resolve the problem is that, without assay standardisation, meta-analyses based on 25(OH)D concentrations are quite simply uninterpretable as it is impossible to pool the disparate results from different studies in any reasonable fashion. Currently, to our knowledge, there are only three meta-analyses based on standardised 25(OH)D levels^(65–67)^, and all were conducted as part of the ODIN project^(68)^. It was suggested recently that unstandardised results *might* end up providing the same answer as standardised data^(69)^. However, given the amount of assay variability that exists, without standardisation, we will *never* know if the results are correct. *In today’s evidence-based world, guidelines and policy simply cannot be made based on serendipity*. In summary, without accurate and precise data, countries with current guidelines committees are stuck in place, and other agencies/medical societies wanting to develop guidelines appear to be selecting one set of guidelines or the other without the data needed to resolve the differences. That means that without accurate and precise data, there is an inadequate basis to advance the vitamin D field and establish if 25(OH)D increases or decreases the risk of non-skeletal diseases. Thus, the paralysis that has ensued expands and worsens.

## VDSP: a ready solution

The National Institutes of Health (NIH) Office of Dietary Supplements (ODS) established the VDSP in 2010 and coordinated its efforts until 2018. Since 2018 it has been coordinated as an independent agency. From the beginning, VDSP has been an international collaborative effort to standardise the laboratory measurement of serum total 25(OH)D and other potential markers of vitamin D status in order to improve clinical and public health practice^(6,70)^.

A standardised laboratory measurement is defined as one that provides the ‘true’ total 25(OH)D concentration as measured by the three JCTLM-recognised reference measurement procedures^(71)^. Serum 25(OH)D measurements can be ‘prospectively’ standardised using a standardised assay, or they can be ‘retrospectively’ standardised, after the fact, using methods developed by the VDSP^(14,72,73)^.

Two examples from representative national surveys highlight the importance of having standardised 25(OH)D data to evaluate current levels, trends and seasonal differences in both representative national health surveys and vitamin D research. The first example is from the US National Health and Nutrition Examination Surveys (NHANES). In four surveys from 1988 to 2006, there appeared to be a dramatic drop in mean serum 25(OH)D levels for all persons aged >12 based on the original assay measurements (Fig. 4). DiaSorin Radioimmunoassay was used originally in all four surveys. At the time it could not be determined if the trend was real or not. However, when VDSP methods were used to retrospectively standardise the results for all four surveys, it became clear that the ‘decline’ was an assay artefact^(74)^.

**Fig. 5:**
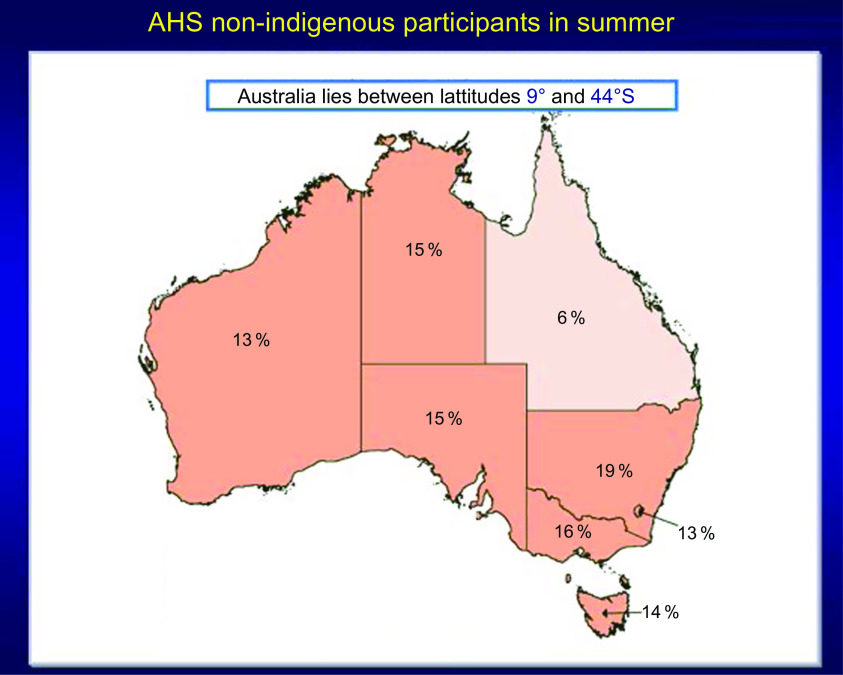
Prevalence of vitamin D deficiency in Australian summer months (serum 25(OH)D concentration <20 ng/ml (50 nmol/l); survey weighting factors were used to calculate representative prevalence figures for each state or territory). Australian Health Survey non-indigenous participants by state and territory, Australia, 2011–2012. Source: Australian Bureau of Statistics^(75)^ (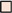, 10 %; 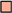, 10 to <20 %; 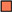, 20 to <30 %; 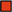, 30 to <40 %; 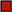, 40 to <50 %)

**Fig. 6:**
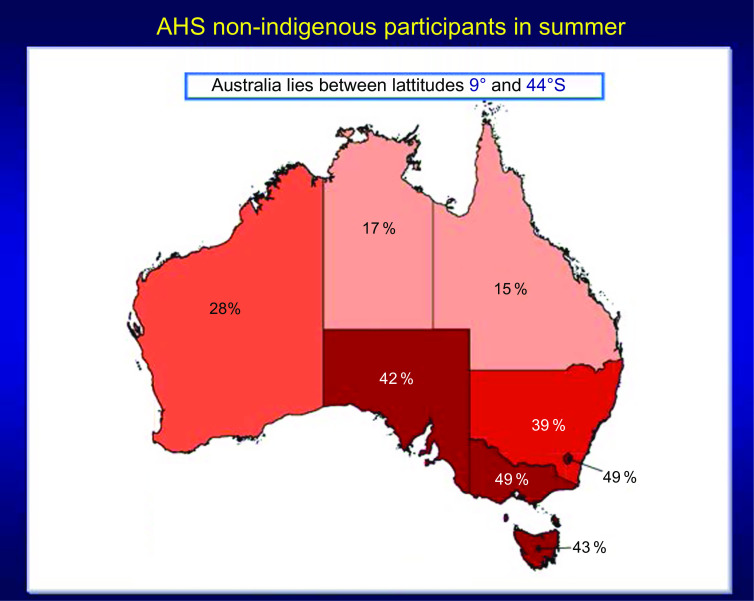
Prevalence of vitamin D deficiency in Australian winter months (serum 25(OH)D concentration <20 ng/ml (50 nmol/l); survey weighting factors were used to calculate representative prevalence figures for each state or territory). Australian Health Survey non-indigenous participants by state and territory. Australia, 2011–2012. Source: Australian Bureau of Statistics^(75)^ (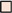, 10 %; 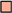, 10 to <20 %; 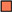, 20 to <30 %; 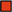, 30 to <40 %; 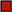, 40 to <50 %)

The second example is from the 2011–2012 Australian Health Survey (AHS) where it was found that the prevalence of vitamin D deficiency, that is, <20 ng/ml or 50 nmol/l, increased *dramatically* in winter months compared to summer months^(75,76)^ (Figs 5 and 6). Because serum 25(OH)D was measured with a prospective VDSP-standardised LC-MS/MS assay, it is now possible for national and state governments to develop policy and cost estimates for programmes to ameliorate the situation based on accurate and precise estimates of current 25(OH)D levels and the prevalence of deficiency by region, age, sex, ethnic group and other potential risk factors^(77)^. Countries without representative standardised 25(OH)D data can only guess in the development of vitamin D policy, programmes and their costs, which can further lead to a loss of political support for national surveys.

Two important questions are then: (1) how to select an assay; and (2) what are the key phases in VDSP.

VDSP criteria for selecting an assay are the following:

1. Fit for use;
2. Certified by the CDC Vitamin D Standardization Certification Program as being standardised^(78)^ and having an appropriate measurement range or be a documented standardised laboratory-developed HPLC or LC-MS/MS assay with an appropriate measurement range;
3. Appropriate level of assay precision and accuracy; and
4. Meets VDSP assay standardisation criteria in your ‘hands’ or laboratory.

‘Fit for use’ is a new criterion and has been added with the realisation that some immunoassays do not function appropriately in all patient populations^(79)^. It means that the assay chosen will perform appropriately and provide standardised measurements in the patient/study populations in the conditions for which it will be used. This criterion applies solely to non-chromatography-based immunoassays. Depending on the situation, you may need an assay that measures serum 25(OH)D_2_ and 25(OH)D_3_ accurately, or you may need one that measures total 25(OH)D in specific conditions, for example, pregnancy or in different pathophysiological states. Importantly, some immunoassays do not function well in pregnant women or in people with different diseases^(79)^. Additionally, data suggest that some immunoassays do not measure serum 25(OH)D_2_ well^(79,80)^. This can be a problem when ergocalciferol (vitamin D_2_) is used to treat vitamin D deficiency, as is the case in the USA or is used by vegetarians. It is apparent that if an assay cannot be verified to be fit for use, it should not be utilised.

If you are planning to select an immunoassay, we suggest that you first see which ones are currently, or have been in the past, certified by the CDC as meeting VDSP performance criteria of having a total CV ≤ 10 % and a mean bias with the range of –5 to +5 %^(72,74)^. The list of current and past CDC-certified assays is available from the CDC website: https://www.cdc.gov/labstandards/hs.html.

The CDC list also gives information on the assays’ stated measurement range. VDSP recommends using an assay that does have an appropriate measurement range for the population it will be used in; for example, it should be able to measure 25(OH)D in persons who are deficient.

Representative national nutrition surveys need to have the very highest level of accuracy and precision. Therefore, it has been recommended that a standardised LC-MS/MS assay be selected^(81)^.

There is another reason for national health surveys and researchers to consider using an LC-MS/MS assay. At present, the vitamin D field is in a great deal of flux where additional vitamin D metabolites, for example, 3-epi-25(OH)D_3_, 24R,25(OH)_2_D_3_ and vitamin D-binding protein (VDBP), may turn out to be essential to assessing vitamin D status^(82,83)^. For national health surveys to maintain political support, they need to be flexible enough to respond to the needs that were not anticipated at the time the survey was designed. As a result, we suggest that, if possible, it would be prudent to measure 25(OH)D_2_, 25(OH)D_3_, 3-epi-25(OH)D_3_, 24R,25(OH)_2_D_3_ and possibly VDBP as well in those surveys. Given that researchers around the world are generally working with very limited budgets, we suggest that research grant applications include measurement of those compounds where it fits in with the hypotheses being tested and, otherwise, request funds to collect and appropriately store serum samples for potential future analyses.

It is possible to standardise the measurements for those analytes and 25(OH)D to 24R,25(OH)_2_D_3_ ratio and VDBP as well given that there are reference methods and reference materials available^(27,84–86)^. NIST SRMs 972a, 1949 and 2973 along with selected DEQAS samples provide target values for 25(OH)D_2_, 25(OH)D_3_, 3-epi-25(OH)D_3_ and 24R,25(OH)_2_D_3_^(14,87,88)^. NIST SRM 1949 includes target values for VDBP (Table 4)^(88)^. However, at present, VDSP statistical criteria to define standardisation/traceability for those compounds have not been defined. Currently, the VDSP and the International Federation of Clinical Chemistry and Laboratory Medicine are collaborating to define those statistical criteria.

**Table 4:** Reference values for Standard Reference Material® 1949: Frozen Human Prenatal Serum*

Constituent†	Group							
	Non-pregnant		First trimester		Second trimester		Third trimester	
	Mean	Expanded uncertainty	Mean	Expanded uncertainty	Mean	Expanded uncertainty	Mean	Expanded uncertainty
25(OH)D_3_ (ng/ml)	24·98	0·28	26·01	0·22	30·00	0·50	29·43	0·41
25(OH)D_2_ (ng/ml)	N/A‡		N/A		0·514	0·037	0·897	0·057
3-epi-25(OH)D_3_ (ng/ml)	1·32	0·06	1·20	0·05	1·87	0·07	1·87	0·04
VDBP (μg/ml)	211·5	2·8	286·7	3·8	349·7	4·3	383·4	5·1
VDBP (μmol/kg)	4·01	0·05	5·43	0·06	6·64	0·07	7·28	0·08

3-epi-25(OH)D_3_, 3-epi-25-hydroxyvitamin D_3_; VDBP, vitamin D-binding protein.

* Additional target values given for measures of thyroid function, as well as for copper, selenium and zinc. For additional details, see National Institute for Standards and Technology. Certificate of Analysis Standard Reference Material® 1949: Frozen Human Prenatal Serum.

† Equations to convert from ng/ml to nmol/l: (1) 25(OH)D_3_: 2·496 × ng/ml; (2) 25(OH)D_2_: 2·423 × ng/ml; (3) 3-epi-25(OH)D_3_: 2·496 × ng/ml.

‡ No concentration value provided in the Certificate of Analysis.

To accomplish assay standardisation, we recommend the following two-phase approach:

- Phase 1: verification of fit for use
- Phase 2: calibration of assay to meet VDSP criteria, that is, total CV < 10 % and mean bias ± 5 %^(14,72)^.

Verification of fit for use can be accomplished by testing the assay against a VDSP standardised LC-MS/MS assay. On the other hand, NIST SRM 1949 includes reference measurement target values in sera from pregnant women in the first, second and third trimesters and should be used to verify that the intended assay is ‘fit for use’ in pregnant women (Table 4)^(88)^.

An essential point that needs repeating is that laboratories cannot assume that just because an immunoassay assay is CDC-certified it will function appropriately in their hands^(89,90)^. We recommend a testing period in order to verify that an immunoassay is standardised especially since there is generally very little an individual laboratory can do to ‘calibrate’ an immunoassay.

**Fig. 7:**
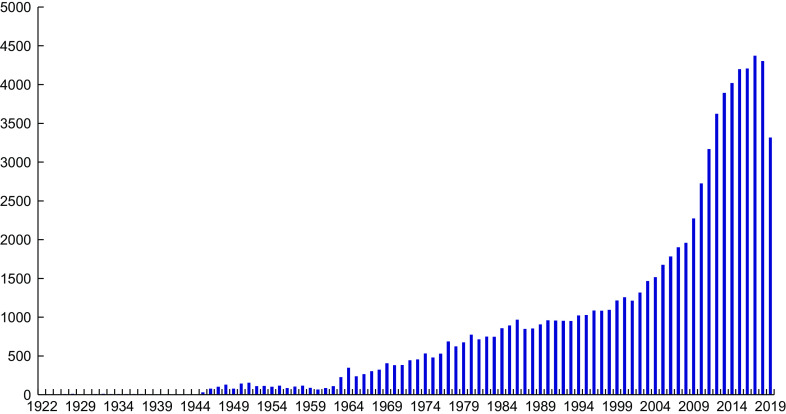
Vitamin D papers published since 1922. Source: PubMed: https://www.ncbi.nlm.nih.gov/pubmed/ (accessed September 2019)

If we started today to conduct all vitamin D research using only assays that were both ‘fit for use’ and VDSP-standardised, would we be able to decide between IOM 2011 and Endocrine Society guidelines (Table 3)? The sad fact is that we could not, until substantial amounts of new, standardised data are available. Since the discovery of serum 25OHD_3_ in 1968^(91)^, approximately 80 000 vitamin D papers have been published (Fig. 7). Virtually all of those, where serum total 25(OH)D was measured, reported only unstandardised data. As a result, we recommend that the vitamin D field needs to work with researchers, journal editors and research funding agencies around the world to identify and promote keys studies for *retrospective* standardisation using methods and procedures developed by the VDSP^(73,92)^. At that point, the retrospectively standardised data could be re-analysed and published in meta-analyses based solely on standardised results^(93)^.

Importantly, retrospective standardisation can be accomplished relatively inexpensively as shown in the re-analyses of the Canadian Health Measures and HunMen study data^(12,94)^. Such approaches could be utilised in virtually all vitamin D research. Journal editors are encouraged to require either ‘prospectively’ standardised or ‘retrospectively’ standardised 25(OH)D data as a condition for publication.

We recognise that all science evolves. Similarly, there are weaknesses in the current VDSP performance criteria that will, hopefully, be improved as the vitamin D field progresses. One worthy of brief mention is the VDSP performance criteria for mean bias^(74)^. Nonetheless, we believe it is past time to implement the recommendations laid out here.

## Conclusions

It is past time for the vitamin D research field to embrace reporting only standardised 25(OH)D data. The assays utilised must be: (1) fit for use in the population studied; and (2) standardised (either prospectively or retrospectively) across the appropriate measurement range. Moreover, funding agencies, for example, the US NIH, and journals must make this essential for study funding and publication. Failure to do so will perpetuate the current paralysis and preclude moving the vitamin D field forward.
